# Bibliometric Analysis of Research Trends and Key Areas Related to the Relationship Between Parkinson’s Disease and Exercise Therapy

**DOI:** 10.1155/padi/2781558

**Published:** 2026-09-02

**Authors:** Zengkun Fang, Shuang Zeng, Dingwen Xu, Huachun Liu

**Affiliations:** ^1^ Department of Physical Therapy, Jiangbin Hospital of Guangxi Zhuang Autonomous Region, Nanning 530000, China; ^2^ Department of Rehabilitation Medicine, The First Affiliated Hospital of Guangxi University of Chinese Medicine, Nanning 537500, China, gxtcm.com; ^3^ Department of Clinic, School of Medicine, Yangzhou Polytechnic University, Yangzhou 225100, China

**Keywords:** bibliometric analysis, exercise therapy, key areas, Parkinson’s disease, trends

## Abstract

**Background:**

Exercise therapy is recognized as a crucial method that can markedly improve outcomes for individuals with Parkinson’s Disease. Research on exercise therapy and Parkinson’s Disease is steadily increasing; however, a bibliometric analysis in this field is currently lacking. This study applies bibliometric methods to identify trends and hotspots in research related to Parkinson’s Disease and exercise therapy.

**Methods:**

Data collection comprised articles relevant to Parkinson’s Disease and exercise therapy, which were sourced from the Web of Science core collection for the period between January 1, 2004, and December 31, 2024. The collected literature was subsequently analyzed using a suite of bibliometric tools, including Excel, VOSviewer, and CiteSpace. Additionally, text mining was carried out via Coremine to identify significant correlations among key terms within this research domain.

**Results:**

The volume of articles on Parkinson’s Disease and exercise therapy has increased yearly, with a notable surge after 2019. The top three countries contributing to this volume are the United States, Italy, and China. The leading institutions in publication output are Radboud University Nijmegen, the University of Sydney, and Northwestern University. The 10 most frequent keywords include Parkinson’s Disease, exercise, rehabilitation, people, gait, balance, quality of life, physical therapy, therapy, and motor. Keywords such as telemedicine, telehealth, neurodegeneration, telerehabilitation, and wearable sensors represent current hotspots in this area of research.

**Conclusion:**

This study utilizes bibliometric techniques to perform an objective, quantitative assessment of the literature pertaining to Parkinson’s Disease and exercise therapy. It outlines the current research landscape, pinpoints emerging hotspots, and addresses prevailing challenges, thereby offering valuable insights to guide and improve the focus and efficacy of subsequent studies.

## 1. Introduction

Parkinson’s Disease (PD) is a prevalent neurodegenerative disorder with major motor impairments, creating a clinical demand for exercise interventions [1, 2]. The postural instability and falls associated with PD impose significant healthcare and economic burdens, necessitating exercise interventions aimed at improving these impairments, especially in the early stages of the disease [3].

Current treatments for PD mainly include pharmacotherapy, surgical interventions, and stem cell transplantation, all of which fail to effectively address the motor balance issues faced by PD patients or reduce the incidence of falls, resulting in potential serious complications. Given the suboptimal efficacy of pharmacological and surgical treatments for PD, the Evidence‐Based Medicine Expert Group of the Movement Disorders Society recommends exercise and physical therapy as effective adjuncts to drug therapy [4–6].

In recent decades, research investigating exercise therapy for PD has expanded exponentially, generating a vast and complex body of literature. Although research on PD and exercise therapy is plentiful, the field lacks a comprehensive bibliometric review. While prior bibliometric efforts on this topic either covered shorter windows, focused on overlaying systematic reviews rather than primary articles, or relied on a single visualization toolkit, our study delivers the first 21‐year (2004–2024) quantitative map of the primary literature retrieved from the Web of Science Core Collection and triangulated with Coremine medical text mining to surface biomedical‐entity links beyond keyword co‐occurrence. This gap makes it challenging to map the intellectual landscape, identify emerging trends, and forecast future directions through objective data. Specifically, there is a need to systematically analyze publication trends, key contributing countries/institutions, influential journals and authors, research hotspots, and evolving frontiers within this interdisciplinary field. To address this, our study employs bibliometric methods to analyze relevant publications from the Web of Science Core Collection. The aim is to illuminate research trajectories and provide scholars with a clear understanding of advancements, thereby promoting the more effective application of exercise therapy in PD management. The findings are expected to offer a data‐driven overview, highlight knowledge gaps, and inform future research priorities and collaborative opportunities.

## 2. Methods

### 2.1. Literature Search and Selection Strategy

**FIGURE 1:**
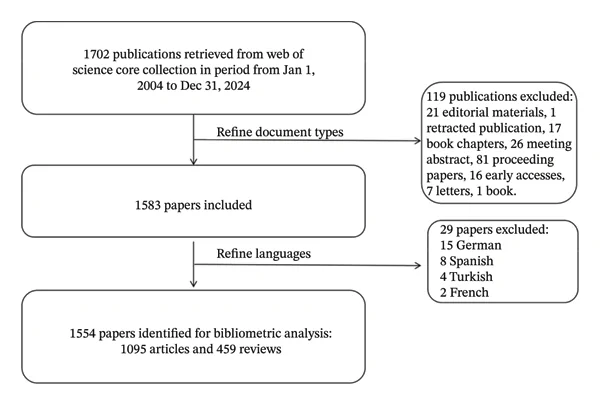
Schematic diagram of the literature search and selection process.

Search the Web of Science Core Collection database with a time frame from January 1, 2004, to December 31, 2024. The search terms include TS = (Exercise Therapy OR (Rehabilitation Exercise) OR (Exercise, Rehabilitation) OR (Exercises, Rehabilitation) OR (Rehabilitation Exercises) OR (Therapy, Exercise) OR (Exercise Therapies) OR (Therapies, Exercise) OR (Remedial Exercise) OR (Exercise, Remedial) OR (Exercises, Remedial) OR (Remedial Exercises)) AND TS = (Parkinson Disease OR (Idiopathic Parkinson’s Disease) OR (Lewy Body Parkinson’s Disease) OR (Parkinson’s Disease, Idiopathic) OR (Parkinson’s Disease, Lewy Body) OR (Paralysis Agitans) OR (Parkinson’s Disease) OR (Idiopathic Parkinson Disease) OR (Lewy Body Parkinson Disease) OR (Primary Parkinsonism) OR (Parkinsonism, Primary) OR (Parkinson Disease, Idiopathic)). The criteria for including and excluding literature were defined based on established methodologies from prior research [7]. The inclusion criteria stipulated that documents must be of the type “article” or “review” and published in English. Conversely, materials such as Editorial Material, News Items, Corrections, Meeting Abstracts, Early Access publications, Letters, and Book Chapters were excluded. Following the application of these filters, the relevant records were exported as a plain text file. To mitigate subjective bias, two authors independently performed the literature selection and data extraction. Any disagreements were resolved through consultation with a third author to reach a consensus (Figure 1).

### 2.2. Bibliometric Analysis

A suite of tools was employed for the bibliometric and visualization analyses, including Excel, an online bibliometric platform (https://bibliometric.com/), VOSviewer (v1.6.19), and CiteSpace (v.6.1.R6) [8, 9]. Specifically, Microsoft Excel 2019 was utilized to calculate the annual publication counts. The online platform assisted in evaluating the yearly output of high‐productivity countries. National, institutional, and author collaboration networks were mapped using VOSviewer, while CiteSpace was applied to perform cluster analysis and burst detection [7]. CiteSpace was applied to perform cluster analysis and burst detection. The parameters for CiteSpace were set as follows: the time slicing was from January 2004 to December 2024 with 1‐year per slice; the term source was selected from titles, abstracts, author keywords, and keywords plus; the node types were set to “Reference” for co‐citation analysis and “Keyword” for burst detection; the selection criteria for each slice was set to the top 50 most cited or occurring items; and the pruning method used was Pathfinder to simplify the network while preserving its most salient structural links. All remaining CiteSpace settings were kept at their default values to ensure maximum reproducibility.

### 2.3. Text Mining Analysis

Text mining was performed using the online biomedical literature analysis tool Coremine Medical (https://coremine.com/medical/), a validated platform that integrates the Medical Literature Analysis and Retrieval System Online (MEDLINE) database. The tool was used to identify and analyze key biomedical terms significantly associated with both exercise therapy and PD. To ensure the robustness and specificity of the identified associations, we applied a significance threshold of *p* < 0.05 for the “Filter by connection relevance” criterion. In the resulting network visualization, connection lines between nodes represent statistically significant correlations. A thicker line indicates a stronger degree of association. Only entities belonging to the Chemicals, Symptoms, and Procedures ontologies were retained for downstream interpretation to maintain biological relevance and comparability across studies.

## 3. Results

### 3.1. Analysis of Annual Publications

**FIGURE 2:**
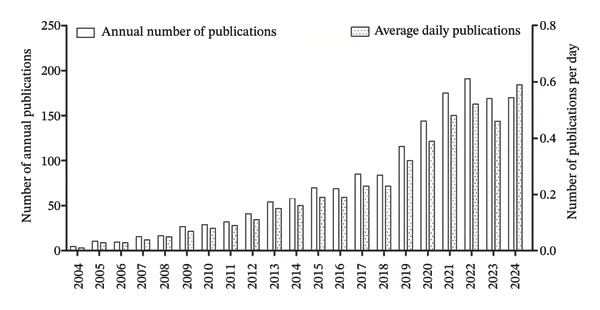
Analysis of annual publications.

From January 1, 2004, to December 31, 2024, a total of 1554 articles meeting our selection criteria were published, consisting of 1095 articles and 459 reviews, as shown in Figure 1. Before 2019, the number of publications each year was below 100. However, after 2019, there was a sharp increase, with 2022 reaching 191 articles, double that of 2018. There was a slight decline in 2023; nonetheless, in 2024, the publication count has again approached 170 articles. We found that in 2004, there was an average of 0.01 articles per day, rising to 0.52 in 2022 and 0.46 in 2023, with an average of 0.59 articles per day in 2024. Therefore, despite occasional fluctuations, articles on PD and exercise therapy show an overall annual increase, particularly evident after 2019 (Figure 2).

### 3.2. Analysis of Countries/Regions

**FIGURE 3:**
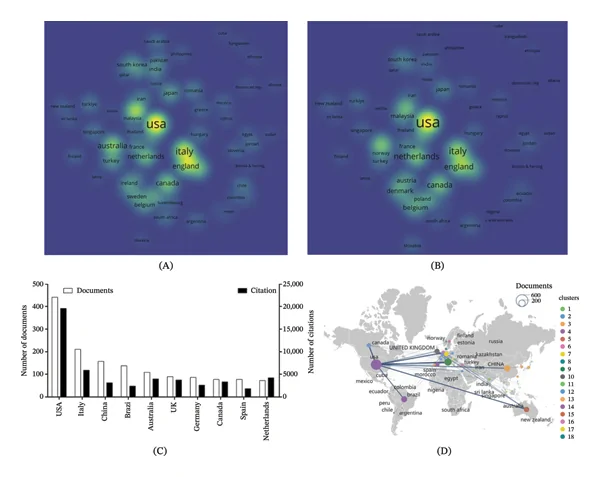
Analysis of countries. (A) Density visualization views based on document numbers. (B) Density visualization views based on citation numbers. (C) Top 10 countries by publication volume. (D) International collaboration network among countries/regions, where node size represents productivity and line thickness indicates collaboration strength.

A total of 75 countries/regions contributed to this field. The number of publications from each country and the citation counts can be illustrated using density visualization views (Figure 3A,B). The top 10 countries ranked by publication volume are shown in Figure 3C, with the United States leading significantly with 442 publications, followed by Italy (*n* = 211) and China (*n* = 156). In terms of total citation counts, the top three countries are the United States (*n* = 19,569), Italy (*n* = 5873), and the Netherlands (*n* = 4194). A co‐authorship analysis of these countries reveals that the highest levels of collaboration come from the United States (TLS = 217), Italy (TLS = 123), and the United Kingdom (TLS = 112). The United States collaborates on published papers with all of the top 10 countries or regions by publication volume (Figure 3C,D).

### 3.3. Analysis of Institutions

**FIGURE 4:**
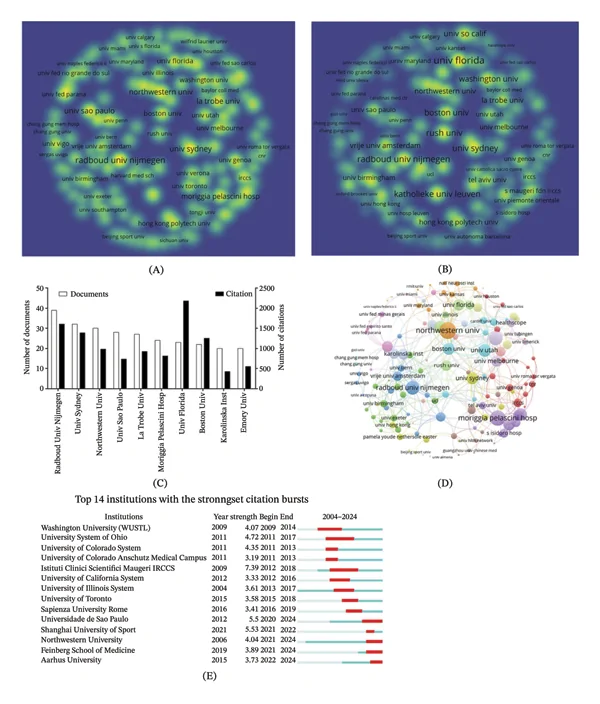
Analysis of institutions. (A) Density visualization views based on document numbers. (B) Density visualization views based on citation numbers. (C) Top 10 institutions by publication volume. (D) Network visualization view of institutional collaboration. (E) Citation burst detection identifying institutions that gained sudden prominence.

A total of 2346 institutions published articles on PD and exercise therapy. Among them, 151 institutions contributed more than five articles. A co‐authorship analysis visualized these 151 institutions, generating density visualization views based on publication volume and citation counts (Figure 4A,B). The top 10 institutions based on publication volume included Radboud University Nijmegen, which published the highest number of articles (39), followed by the University of Sydney (*n* = 32) and Northwestern University (*n* = 30). Although the University of Florida only published 23 articles, it achieved the highest citation count (Figure 4C). Next, the network visualization view revealed a tight collaborative network among these 151 prolific institutions, particularly between Northwestern University (TLS = 46), Moriggia Pelascini Hospital (TLS = 41), and La Trobe University (TLS = 35), indicating strong collaborative relationships with other institutions (Figure 4D). Burst analysis of institutions showed that 14 institutions experienced citation bursts, with Istituti Clinici Scientifici Maugeri IRCCS exhibiting the strongest burst strength (strength = 7.39) and the longest duration (2012–2018). Currently, Universidade de São Paulo, Northwestern University, Feinberg School of Medicine, and Aarhus University are in a period of citation bursts (Figure 4E).

### 3.4. Analysis of Authors

**FIGURE 5:**
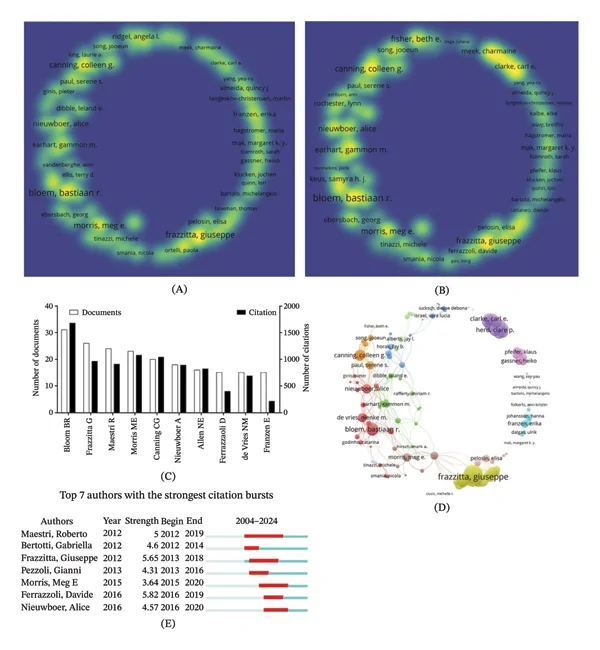
Analysis of authors. (A) Density visualization views based on document numbers. (B) Density visualization views based on citation numbers. (C) Top 10 authors by publication volume. (D) Network visualization view of authors’ collaboration. (E) Citation burst detection for authors.

A total of 6801 authors contributed articles on PD and exercise therapy, with 120 authors having more than five publications. We conducted a co‐authorship analysis on these 150 authors, visualizing their output and citation metrics using density visualization views (Figure 5A,B). The top 10 authors ranked by output include Bloem BR from Radboud University Medical Center, who leads with 31 papers and the highest citation count (*n* = 1676) (Figure 5C). We represent the collaboration among these prolific authors with a network visualization view. Overall, collaboration is tight within groups, but limited between them. For instance, the two highest‐producing authors, Bloem BR and Frazzitta G, show a lack of intergroup collaboration (Figure 5D). Burst analysis reveals seven authors experienced citation bursts, with Ferrazzoli, Davide showing the strongest burst (strength = 5.82) and Maestri, Roberto having the longest burst duration from 2012 to 2019 (Figure 5E).

### 3.5. Analysis of Journals

**FIGURE 6:**
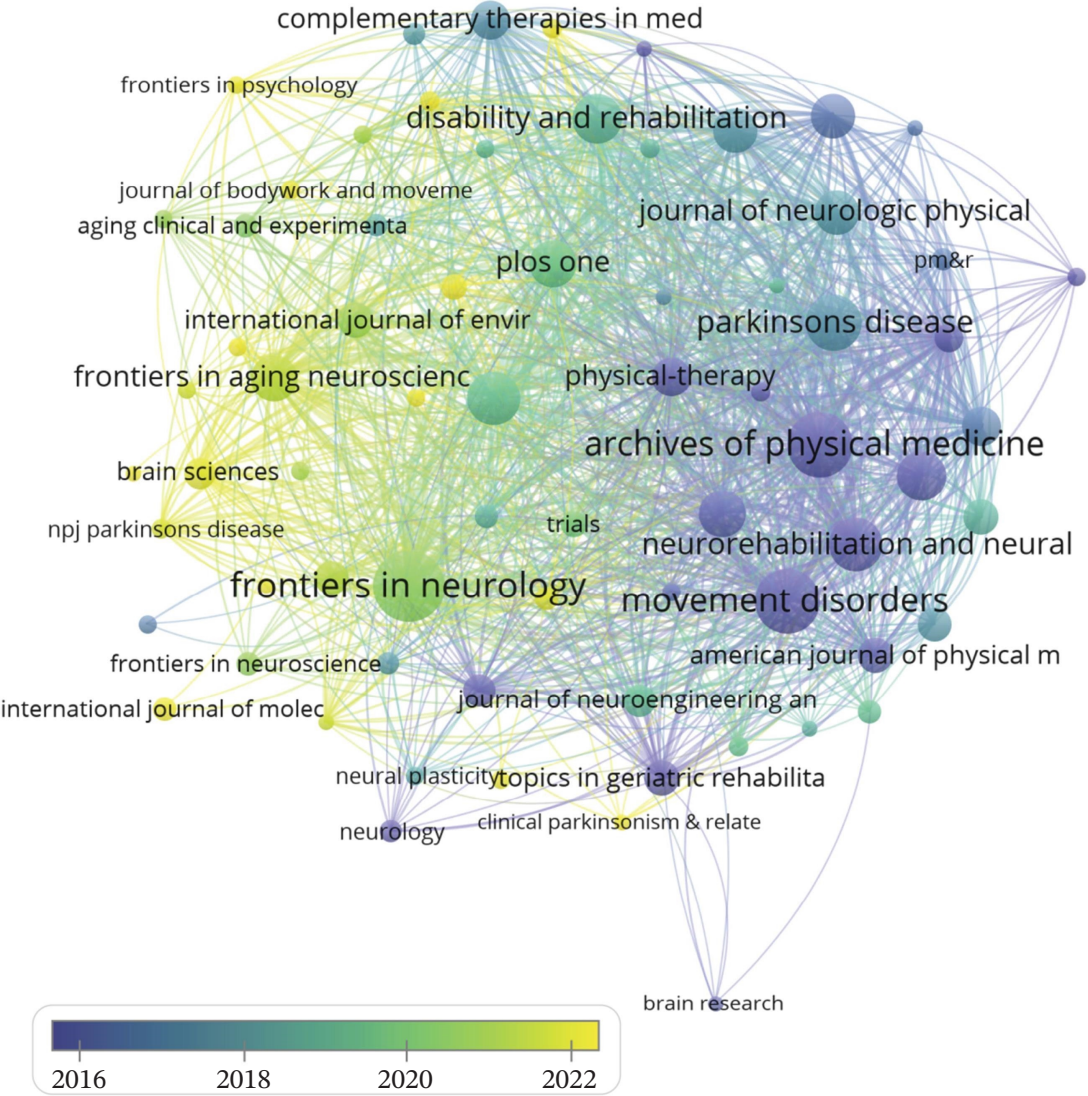
The overlay visualization of bibliographic coupling among journals.

A total of 470 journals contributed articles on PD and exercise therapy, with 70 journals publishing more than five articles. Frontiers in Neurology had the highest publication volume, totaling 52 articles, followed by Archives of Physical Medicine and Rehabilitation (45 articles) and Movement Disorders (44 articles). These three journals had citation counts of 722, 2368, and 4748, respectively. The top 10 journals by publication volume are shown in Table 1, with Movement Disorders having the highest impact factor of 7.4. Four journals are classified as Q1 in JCR, five are Q2, and one is Q3. A bibliometric coupling analysis using VOSviewer on journals with more than five publications revealed that Archives of Physical Medicine and Rehabilitation, Movement Disorders, and Neurorehabilitation and Neural Repair had substantial early publication volumes, while Brain Sciences and NPJ PD had higher publication volumes in recent times (Figure 6).

**TABLE 1:** Top 10 journals by publication volume.

Source	Documents	Citations	Impact factor (2023)	JCR
Frontiers in neurology	52	722	2.7	Q2
Archives of physical medicine and rehabilitation	45	2368	3.6	Q1
Movement disorders	44	4748	7.4	Q1
Parkinson’s disease	35	741	2.1	Q3
Journal of Parkinson’s disease	32	807	4	Q2
Neurorehabilitation and neural repair	32	2219	3.7	Q1
Disability and rehabilitation	29	704	2.1	Q1
Neurorehabilitation	29	617	1.7	Q2
Frontiers in aging neuroscience	27	289	4.1	Q2
Parkinsonism and related disorders	27	1397	3.1	Q2

### 3.6. Analysis of References

**FIGURE 7:**
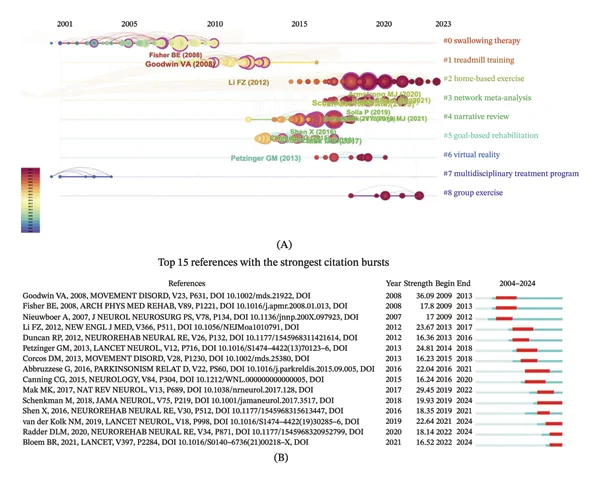
Evolution and landmark references analysis. (A) Timeline visualization of co‐citation clusters, tracking the thematic evolution of the field over time. (B) Top references with the strongest citation bursts, indicating publications that attracted sharp increases in attention.

Table 2 lists the top 10 most frequently cited references in studies regarding PD and exercise therapy. The article with the highest citation count is “Effectiveness of home‐based and remotely supervised aerobic exercise in PD: a double‐blind, randomised controlled trial,” authored by van der Kolk et al., published in 2019 in LANCET NEUROL [10], with 94 citations. Following closely is “Long‐term effects of exercise and physical therapy in people with Parkinson disease,” published by Mak et al. in NAT REV NEUROL in 2017 [11], which has 86 citations. The paper by Schenkman et al., “Effect of High‐Intensity Treadmill Exercise on Motor Symptoms in Patients With De Novo Parkinson Disease: A Phase 2 Randomized Clinical Trial,” published in JAMA NEUROL in 2018 [12], garnered 85 citations. A co‐citation visualization analysis using CiteSpace categorized the co‐cited references into several groups, including swallowing therapy, treadmill training, home‐based exercise, network meta‐analysis, narrative review, goal‐based rehabilitation, virtual reality, multidisciplinary treatment program, and group exercise, totaling nine clusters. The timeline view reflects the evolution of co‐cited references over time (Figure 7A). It shows that citations related to swallowing therapy, treadmill training, and multidisciplinary treatment program were more prevalent prior to 2015. Conversely, citations linked to home‐based exercise, network meta‐analysis, narrative review, goal‐based rehabilitation, virtual reality, and group exercise have become prominent after 2015, indicating currently trending research areas. The burst analysis of references revealed a total of 118 citations discovered, with the top 15 displayed in Figure 7B. The article with the strongest citation burst is “The effectiveness of exercise interventions for people with PD: a systematic review and meta‐analysis,” by Goodwin et al., published in MOVEMENT DISORD in 2008 [13] (Strength = 36.09). Additionally, the 2019 article by van der Kolk et al. in LANCET NEUROL [10], the 2020 paper by Radder et al. in NEUROREHAB NEURAL RE [14], and the 2021 publication by Bloem et al. in LANCET [15] are all still in their citation burst phase.

**TABLE 2:** Top 10 cited references by citations.

Title	Journal	First author	Year	Total citation frequency
Effectiveness of home‐based and remotely supervised aerobic exercise in Parkinson’s disease: a double‐blind, randomised controlled trial	LANCET NEUROL	van der Kolk NM	2019	94
Long‐term effects of exercise and physical therapy in people with Parkinson disease	NAT REV NEUROL	Mak MK	2017	86
Effect of High‐Intensity Treadmill Exercise on Motor Symptoms in Patients With De Novo Parkinson Disease: A Phase 2 Randomized Clinical Trial	JAMA NEUROL	Schenkman M	2018	85
Rehabilitation for Parkinson’s disease: Current outlook and future challenges	PARKINSONISM RELAT D	Abbruzzese G	2016	71
The effectiveness of exercise interventions for people with Parkinson’s disease: a systematic review and meta‐analysis	MOVEMENT DISORD	Goodwin VA	2008	70
Physiotherapy in Parkinson’s Disease: A Meta‐Analysis of Present Treatment Modalities	NEUROREHAB NEURAL RE	Radder DLM	2020	57
Exercise‐enhanced neuroplasticity targeting motor and cognitive circuitry in Parkinson’s disease	LANCET NEUROL	Petzinger GM	2013	52
Diagnosis and Treatment of Parkinson Disease: A Review	JAMA‐J AM MED ASSOC	Armstrong MJ	2020	52
Tai chi and postural stability in patients with Parkinson’s disease	NEW ENGL J MED	Li FZ	2012	51
Effects of Exercise on Falls, Balance, and Gait Ability in Parkinson’s Disease: A Meta‐analysis	NEUROREHAB NEURAL RE	Shen X	2016	41

### 3.7. Analysis of Keywords

**FIGURE 8:**
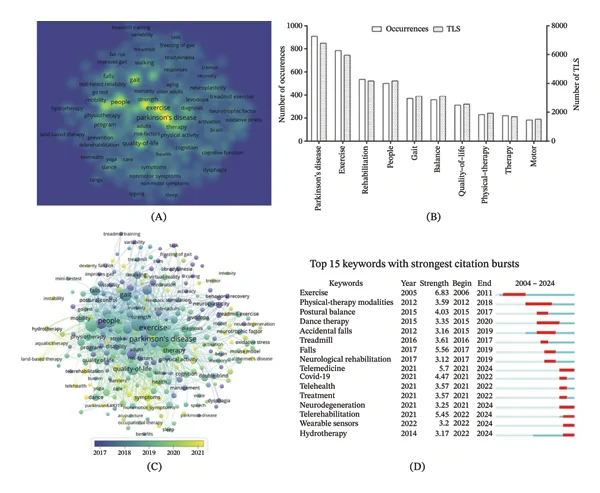
Analysis of keywords. (A) The density visualization of co‐occurrence analysis among keywords. (B) Top 10 keywords by occurrences. (C) Overlay visualization view of keywords. (D) Burst analysis of keywords.

The goal of co‐occurrence analysis is to play a crucial role in monitoring scientific development by exploring popular research directions and fields. Using VOSviewer, keywords that appeared at least 10 times in all papers were selected and analyzed, resulting in the identification of 277 keywords (Figure 8A). The top 10 most frequently occurring keywords are as follows: PD, exercise, rehabilitation, people, gait, balance, quality‐of‐life, physical‐therapy, therapy, and motor (Figure 8B). The overlay visualization view shows that keywords like telerehabilitation, telehealth, and COVID‐19 have emerged in recent years (Figure 8C). Additionally, the bursts analysis of keywords revealed a total of 21 keywords that experienced citation bursts, with the top 15 keywords illustrated in Figure 8D. Among them, exercise exhibited the strongest citation burst (strength = 6.83), and physical‐therapy modalities showed the longest duration of citation bursts (2012–2018). Keywords such as telemedicine, telehealth, neurodegeneration, telerehabilitation, and wearable sensors are still in the citation burst phase (Figure 8D).

### 3.8. Text Mining

**FIGURE 9:**
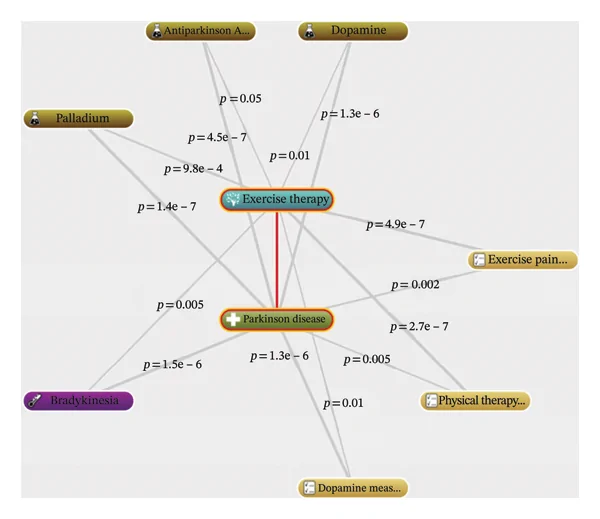
Text mining analysis using coremine.

Using Coremine for text mining reveals significant connections with items related to PD and exercise therapy at the same time (*p* < 0.05). This includes three chemicals: antiparkinson agents, dopamine, and palladium; one symptom: bradykinesia; and three procedures: dopamine measurement, physical therapy exercises, and exercise pain management (Figure 9).

## 4. Discussion

It should be noted that this bibliometric analysis aims to map the intellectual structure and evolving trends of research on exercise therapy for Parkinson’s disease; it does not assess the methodological quality of individual studies nor provide direct evidence regarding the clinical effectiveness or comparative efficacy of specific interventions. The role of exercise therapy in PD has gained widespread recognition among researchers, and studies in this area continue to grow annually, as illustrated in Figure 2 of our article. Given the substantial volume of literature produced and the increasing trend, conducting a bibliometric analysis on the correlation between exercise therapy and PD becomes essential. This will clarify the main themes and directions of current research, highlight existing issues, and identify future research trends. Ultimately, it will provide researchers with a reference point for their investigations, leading to more targeted, efficient, and rational studies.

The United States leads in scholarly output and citation impact within this field, a dominance evident across national, institutional, and researcher levels. This dominance is not merely a product of greater publication volume, but reflects a long‐standing concentration of NIH‐funded neurodegenerative disease research programs, the early establishment of standardized exercise protocols for PD, and the presence of large, multicenter clinical trial networks that consistently generate high‐impact, citable evidence [16]. Furthermore, certain European nations, notably Italy, the Netherlands, and the United Kingdom, exhibit considerable capabilities in scientific output and innovation as well. These countries nurture robust academic and research partnerships with American institutions. Such alliances enhance the synergy among leading research nations. This collaboration between top‐producing countries likely fuels the progressive uptick seen in global research activities. At the same time, the lack of cooperation among countries with relatively low output may also be one reason for their low production levels.

Publication metrics convey a comprehensive overview of the volume of academic output, clearly demonstrating that journals achieving peak publication rates predominantly reside within the Q2 tier or higher, as classified in the Journal Citation Reports (JCR) rankings (Table 1). This trend signifies that investigative research focused on the interplay between PD and exercise therapy commands notable interest from top‐tier scientific publications. Such heightened engagement not only amplifies the visibility of this critical area of study but also propels the evolution of superior research endeavors within the domain. Consequently, this fosters an environment conducive to the generation of groundbreaking and innovative findings, thereby enhancing our collective understanding and treatment modalities related to PD.

A thorough examination of the references and associated keywords reveals that contemporary research trends and hotspots predominantly center on critical areas of study. The analytical framework indicates that the majority of the frequently cited literature primarily focuses on clinical trials (Table 2). This notable observation highlights an urgent and sustained interest in developing effective clinical treatments for PD, particularly regarding the therapeutic efficacy of exercise‐based interventions. Additionally, the burst analysis of references suggests that this area of research continues to garner significant attention, as numerous referenced papers remain in a stage of heightened scholarly activity, signifying ongoing advancements and explorations in this vital field of study. Such insights can guide future inquiries and potentially inform clinical practices aimed at optimizing patient outcomes in the management of PD using exercise‐based interventions.

Keyword analysis reveals that the most frequently occurring terms relate to motor abilities and exercise training in PD. Emerging keywords, such as telemedicine, telehealth, telerehabilitation, and wearable sensors, have surged in recent years and are still experiencing a citation explosion (Figure 8). This lexical shift signals a structural reorientation of the field: the convergence of rehabilitation science with digital health, accelerated by pandemic‐era disruptions to in‐person care and by the clinical imperative to monitor fluctuating PD symptoms outside controlled laboratory settings. Our bibliometric data further reflect this transition, with “artificial intelligence” and “machine learning” appearing as nascent but rapidly growing keyword clusters, suggesting an emerging—though still methodologically heterogeneous—research front rather than an established clinical standard. Telemedicine has provided avenues to meet the ongoing healthcare needs of PD patients, minimizing the need for face‐to‐face outpatient visits and reducing human contact amid a range of infectious diseases, including COVID‐19. Remote monitoring has been widely adopted among PD patients, utilizing dedicated devices, wearable sensors, and mobile applications for assessing symptoms like tremors, bradykinesia, gait, falls, and speech rehabilitation [17–19]. The International Parkinson and Movement Disorder Society (MDS) also offers Parkinson’s exercise training video consultations for African physicians through its “virtual professor program.” [20] However, significant barriers persist, including technological challenges, privacy concerns, regulations, and reimbursement issues, hindering the implementation of these advanced technologies. Addressing these challenges will be essential for harnessing the full potential of telehealth solutions in the realm of PD management [21].

The practical implementation and development of telemedicine in PD care require addressing these challenges through concerted international collaboration. Future efforts should focus on establishing global standards for data interoperability, validating remote assessment tools, and creating evidence‐based frameworks for reimbursement. Moreover, the maturation of the field calls for robust, long‐term studies to evaluate the sustained effectiveness of these digital interventions, as projected by the current trajectory of publication and citation bursts identified in our analysis, ensuring that the promising trend toward technology‐integrated, interdisciplinary research translates into tangible, equitable, and lasting benefits for patients worldwide.

Furthermore, a key contribution of this study lies in the application of the Coremine platform for integrated text mining. This approach moves beyond conventional co‐occurrence analysis to uncover deeper semantic connections between biomedical entities. Through the innovative platform Coremine, we thoroughly analyzed and elucidated a myriad of categories that exhibit substantial interrelations with both PD and exercise therapy. These pivotal categories encompass Chemicals, Symptoms, and Procedures. Our investigation uncovered several critical conclusions that emerged during the rigorous application of exercise therapy aimed at mitigating the effects of PD. Such conclusions provide a foundational framework for researchers, enabling them to conduct more in‐depth explorations of the therapeutic impacts of exercise therapy on individuals diagnosed with Parkinson’s. This multidimensional mapping of the knowledge structure, spanning from foundational clinical evidence to cutting‐edge digital health technologies, offers a holistic and nuanced perspective that is often missing in narrower bibliometric reviews. Furthermore, these insights may also inform the optimization of therapeutic strategies within the context of PD, thereby fostering a broader understanding of neurodegenerative disorders. Hence, the interplay between exercise therapy and these conditions warrants further examination, considering its potential to enhance patient outcomes and quality of life.

In addition, the scope of exercise therapy is expanding beyond conventional physical training. Artistic movement‐based interventions, such as dance and theater, are emerging as a significant frontier in both research and practice. For example, theater training [22] has been shown to effectively improve emotional well‐being and quality of life in PD, while dance is extensively studied for its benefits on motor function, balance, and overall well‐being. These approaches offer unique value in holistic rehabilitation by integrating music, rhythm, social interaction, and emotional expression. This provides a unique and multidimensional perspective for future research.

## 5. Future Research and Policy Recommendations

Future research should prioritize large‐scale, multicenter randomized controlled trials with a minimum 12‐month follow‐up, specifically deploying CE‐marked or FDA‐cleared wearable sensors to track real‐world adherence and motor fluctuations outside clinical settings—addressing a critical evidence gap compared to telerehabilitation maturity in stroke or multiple sclerosis. To advance the field, policymakers and funders should promote interdisciplinary collaboration and streamline regulatory pathways for digital therapeutics. Concretely, agencies such as the NIH and EU Horizon Europe should establish dedicated funding calls for hybrid efficacy‐implementation trials of digital exercise therapies, while regulators should issue unified validation guidance for AI‐driven gait and tremor monitoring algorithms to reduce market‐entry uncertainty. Developing shared data standards and open repositories, akin to initiatives in Alzheimer’s disease research, would facilitate pooled analyses and AI‐driven personalization of exercise interventions.

## 6. Limitations

This study uses the Web of Science database for its bibliometric analysis. Although Web of Science is comprehensive for high‐impact English literature, this choice introduces limitations. Unlike systematic reviews or meta‐analyses, bibliometrics excels at mapping broad research trends but does not deeply evaluate study quality or synthesize findings. Our methodological choice of Web of Science, complemented by text mining from the MEDLINE‐derived Coremine platform, prioritizes citation data consistency and biomedical term standardization. However, this focus may introduce a database bias, as relevant high‐quality studies indexed in other databases (e.g., Scopus, PubMed, or regional databases like China National Knowledge Infrastructure (CNKI)) or published in languages other than English are excluded. This could skew the geographical and linguistic representation of the research field, potentially underrepresenting contributions from non‐English‐speaking regions. The text mining results from Coremine are derived solely from MEDLINE, a limitation that may further narrow the scope. To compensate for these biases, future research could employ a multidatabase strategy (e.g., combining Web of Science, Scopus, and EMBASE) and, where feasible, include non‐English publications to provide a more globally inclusive overview. Additionally, triangulating bibliometric findings with insights from expert qualitative reviews could help validate the identified trends and mitigate the limitations of a purely quantitative approach.

## 7. Conclusions

In summary, this study utilizes bibliometric techniques to perform an objective, quantitative assessment of the literature pertaining to PD and exercise therapy. It outlines the current research landscape, pinpoints emerging hotspots, and addresses prevailing challenges, thereby offering valuable insights to guide and improve the focus and efficacy of subsequent studies.

## Author Contributions

Huachun Liu and Dingwen Xu designed the study. Zengkun Fang performed the analysis using VOSviewer and wrote the draft. Shuang Zeng conducted the analysis using CiteSpace and Coremine.

## Funding

The authors have nothing to report.

## Ethics Statement

The authors have nothing to report.

## Consent

The authors have nothing to report.

## Conflicts of Interest

The authors report no conflicts of interest.

## Data Availability

The data that support the findings of this study are available from the corresponding author upon reasonable request.
